# Supplementary material for: Examining Final-Administered Medication as a Measure of Data Quality: A Comparative Analysis of Death Data with the Central Cancer Registry in Republic of Korea
Source: Cancers (Basel). 2023 Jun 27;15(13):3371. doi: 10.3390/cancers15133371 (PMC10341326; doi:10.3390/cancers15133371)
Supplement: Supplementary file 1 [file cancers-15-03371-s001.zip › cancers-2360954-supplementary.pdf]

# Supplementary Materials: Examining final administered medication as a measure of data quality: A comparative analysis of death data with the Central Cancer Registry in South Korea

**Yae Won Tak, Jeong Hyun Han, Yu Jin Park, Do-Hoon Kim, Ji Seon Oh, and Yura Lee**

**Table S1.** Demographics.

| Variable. | **n=179,747** | **%** |
|---|---|---|
| Gender | | |
| Female | 64,552 | 35.9 |
| Male | 115,195 | 64.1 |
| Age at death | | |
| Up to 59 | 46,651 | 26.0 |
| 60–69 | 48,810 | 27.2 |
| 70–79 | 43,504 | 24.2 |
| 80–89 | 33,993 | 18.9 |
| 90–99 | 6,274 | 3.5 |
| 100+ | 515 | 0.3 |
| Type of death | | |
| Cancer-registered | 128,099 | 71.3 |
| In-hospital | 51,648 | 28.7 |
| Year of death | | |
| 2000 or later | 2,760 | 1.5 |
| 1980–1999 | 6,008 | 3.3 |
| 1960–1979 | 43,106 | 24.0 |
| 1940–1959 | 89,709 | 49.9 |
| 1920–1939 | 35,626 | 19.8 |
| 1900–1919 | 2,524 | 1.4 |
| 1899 or earlier | 14 | 0.0 |
| Final treatment classification code | | |
| Outpatient | 99,800 | 55.5 |
| Inpatient | 61,161 | 34.0 |
| Emergency | 18,763 | 10.4 |
| Medical checkup | 17 | 0.0 |
| Unknown | 6 | 0.0 |

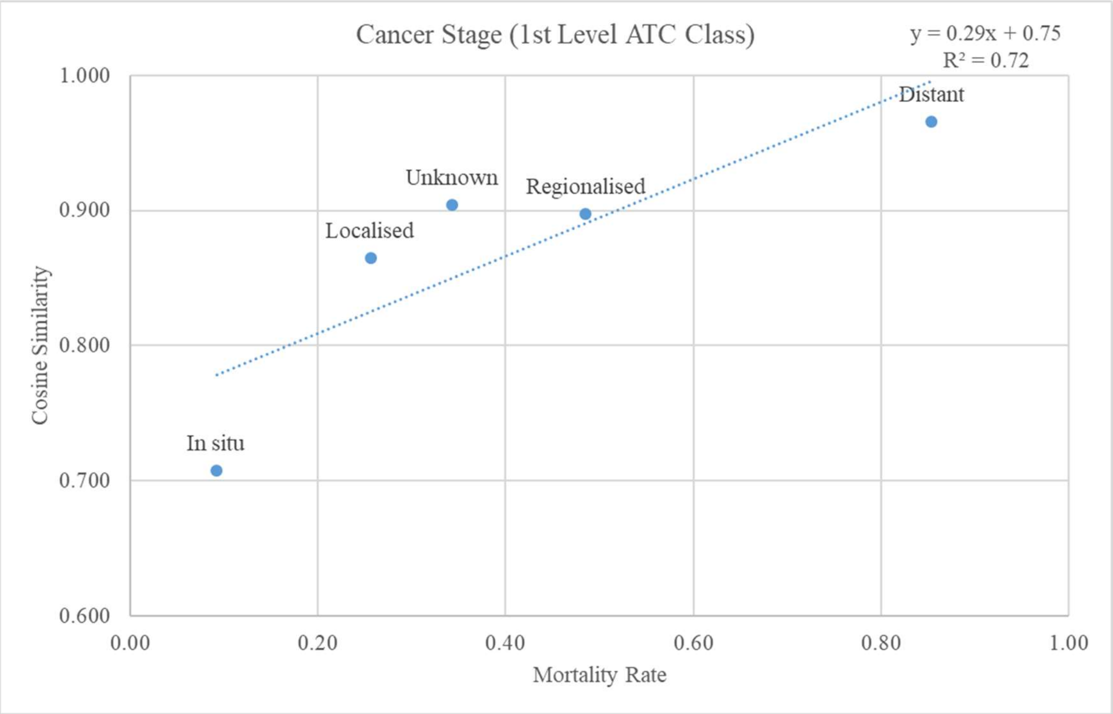


**Figure S1.** Relationship between mortality rate and regrouped cancer stages for the 1st level ATC class.

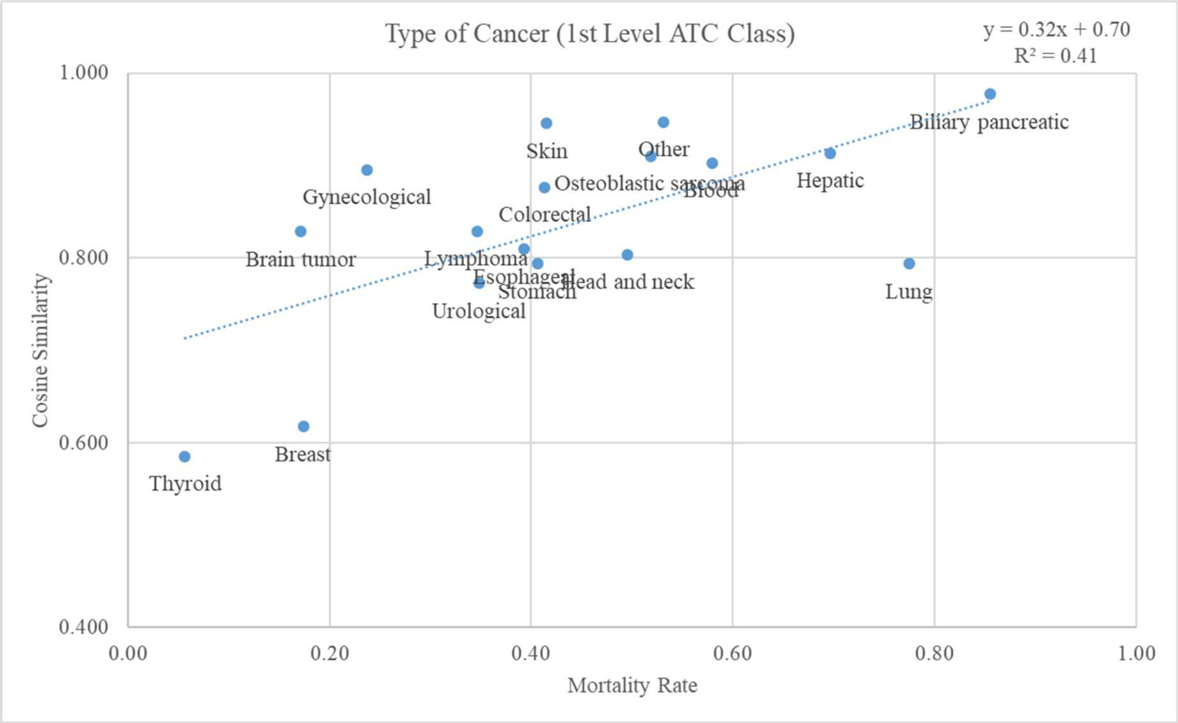


**Figure S2.** Relationship between mortality rate and cancer types for the 1st level ATC class.
